# Comparison of Cyclic Hysteresis Behavior between Cross-Ply C/SiC and SiC/SiC Ceramic-Matrix Composites

**DOI:** 10.3390/ma9010062

**Published:** 2016-01-19

**Authors:** Longbiao Li

**Affiliations:** College of Civil Aviation, Nanjing University of Aeronautics and Astronautics, No. 29 Yudao St., Nanjing 210016, China; llb451@nuaa.edu.cn; Tel.: +86-25-8489-5963

**Keywords:** ceramic-matrix composites (CMCs), cross-ply, hysteresis loops, matrix cracking, interface debonding

## Abstract

In this paper, the comparison of cyclic hysteresis behavior between cross-ply C/SiC and SiC/SiC ceramic-matrix composites (CMCs) has been investigated. The interface slip between fibers and the matrix existed in the matrix cracking mode 3 and mode 5, in which matrix cracking and interface debonding occurred in the 0° plies are considered as the major reason for hysteresis loops of cross-ply CMCs. The hysteresis loops of cross-ply C/SiC and SiC/SiC composites corresponding to different peak stresses have been predicted using present analysis. The damage parameter, *i.e.*, the proportion of matrix cracking mode 3 in the entire matrix cracking modes of the composite, and the hysteresis dissipated energy increase with increasing peak stress. The damage parameter and hysteresis dissipated energy of C/SiC composite under low peak stress are higher than that of SiC/SiC composite; However, at high peak stress, the damage extent inside of cross-ply SiC/SiC composite is higher than that of C/SiC composite as more transverse cracks and matrix cracks connect together.

## 1. Introduction

Nickel-based superalloys with thermal and environmental ceramic coatings are the current load bearing material system that can operate above the metal substrate’s melting temperature, *i.e.*, about 1100 °C, at which a combined-cycle gas turbine will operate at 60% fuel efficiency. However, due to demands for reduced fuel consumption, lighter and hotter engines are required, especially in aviation. Ceramic materials can operate at high temperature with creep resistance, at which metals cannot. However, their use as structural components is severely limited because of their brittleness. Continuous fiber-reinforced ceramic-matrix composites (CMCs), by incorporating fibers in ceramic matrices, however, can be made as strong as metal, yet are much lighter and can withstand much higher temperatures exceeding the capability of current nickel alloys typically used in high-pressure turbines, which can increase the efficiency of aero engines [1]. CMC durability has been validated through ground testing or commercial flight testing in demonstrator or customer gas turbine engines accumulating almost 30,000 h of operation. The CMC combustion chamber and high-pressure turbine components were designed and tested in the ground testing of the GEnx aero engine [2]. The CMC rotating low-pressure turbine blades in a F414 turbofan demonstrator engine were successfully tested for 500 grueling cycles to validate the unprecedented temperature and durability capabilities by GE Aviation (Fairfield, CT, USA). The CMC tail nozzles were designed and fabricated by SNECMA (SAFRAN, Paris, France) and completed the first commercial flight on CFM56-5B aero engine (CFM International, Cincinnati, OH, USA) on 2015. CMCs will play a key role in the performance of CFM’s LEAP (Leading Edge Aviation Propulsion) turbofan engine, which would enter into service in 2016 for Airbus A320 and in 2017 for the Boeing 737 max.

Under cyclic loading and unloading, matrix cracking and fiber/matrix interface debonding occur inside of CMCs [3]. The hysteresis loops appear as the fiber slips relative to matrix in the interface debonded region [4]. The shape, location, and area of hysteresis loops can reveal the internal damage evolution of CMCs subjected to cyclic loading [5]. Many researchers investigated characteristics of hysteresis loops. Kotil *et al*. [6] investigated the effect of interface shear stress on the shape and area of hysteresis loops in unidirectional CMCs. Pryce and Smith [7] investigated the effect of interface partially debonding on hysteresis loops of unidirectional CMCs by assuming purely frictional load transfer between fibers and the matrix. Ahn and Curtin [8] investigated the effect of matrix stochastic cracking on hysteresis loops of unidirectional CMCs and compared with the Pryce-Smith model [7]. Solti *et al*. [9] investigated the effect of interface partially and completely debonding on hysteresis loops in unidirectional CMCs using the maximum interface shear strength criterion to determine interface slip lengths. Vagaggini *et al*. [10] investigated the effect of interface debonded energy on hysteresis loops of unidirectional CMCs based on the Hutchinson-Jensen fiber pull-out model [11]. Cho *et al*. [12] investigated the evolution of interface shear stress under cyclic-fatigue loading from frictional heating measurements. Li *et al*. investigated the effect of interface debonding [13], fibers Poisson contraction [14], fiber fracture [15], and interface wear [16] on hysteresis loops of unidirectional CMCs, and developed an approach to estimate interface shear stress in unidirectional CMCs through hysteresis loop area [17]. Kuo and Chou [18] investigated matrix multicracking in cross-ply CMCs and classified the multiple cracking states into five modes, in which cracking mode 3 and mode 5 involve matrix cracking and interface debonding in the 0° plies.

The objective of this paper is to compare the cyclic hysteresis behavior between cross-ply C/SiC and C/SiC CMCs. The interface slip between fibers and the matrix existed in matrix cracking mode 3 and mode 5, in which matrix cracking and interface debonding occurred in the 0° plies, are considered as the major reason for hysteresis loops of cross-ply CMCs. The hysteresis loops of cross-ply C/SiC and SiC/SiC composites corresponding to different peak stresses have been predicted using present analysis. The differences between C/SiC and SiC/SiC composite on damage parameters and hysteresis dissipated energy have been investigated.

## 2. Materials and Experimental Procedures

### 2.1. Cross-Ply C/SiC Composite

The T-700™ carbon (Toray Institute Inc., Tokyo, Japan) fiber-reinforced silicon carbide matrix composites (C/SiC CMCs) were provided by Shanghai Institute of Ceramics, People’s Republic of China. The fibers have an average diameter of 7 μm and come on a spool as a tow of 12 k fibers. The cross-ply C/SiC composite was manufactured by hot-pressing method, which offered the ability to fabricate dense composite via a liquid phase sintering method at a low temperature. The lay-ups supplied were in the form of (0/90/0/90/0/90/0/90/0). The volume fraction of fibers was about 40%. The void content in the manufactured plates is below 5%. Low pressure chemical vapor infiltration was employed to deposit approximately 5~20 layer PyC/SiC with mean thickness of 0.2 μm in order to enhance the desired non-linear/non-catastrophic tensile behavior.

The dog bone-shaped specimens, with dimensions of 123 mm length, 3.8 mm thickness according to ASTM (American Society for Testing and Materials) standard C 1360-10 [19], and 10 mm width in the gage section of cross-ply C/SiC composite, were cut from 150 mm × 150 mm panels by water cutting. The specimens were further coated with SiC of ~20 μm thick by chemical vapor deposition to prevent oxidation at elevated temperature.

The loading/unloading tensile experiments at room temperature were conducted on an MTS Model 809 servo hydraulic load-frame (MTS System Crop., Minneapolis, MN, USA) equipped with edge-loaded grips, operated at the loading rate of 2.0 MPa/s. The gage-section strains were measured using a clip-on extensometer (Model No. 634.12F-24, MTS Systems Corp.; modified for a 25 mm gage-length). The direct observations of matrix cracking were made using a HiROX optical microscope (Tokyo, Japan). The matrix crack density was determined by counting the number of the cracks in a length of about 15 mm.

### 2.2. Cross-Ply SiC/SiC Composite

The Hi-Nicalon Type S™ fiber reinforced pre-impregnated melt-infiltrated silicon-carbide matrix composites (SiC/SiC CMC) were provided by GE Aviation (Cincinnati, OH, USA) [20]. The specimens were machined to a dogbone shape with dimensions of 203 mm length, 10.16 mm width, and 1.88 mm thickness. The lay-ups supplied were in the form of [0/90]_2s_. During the final phase of manufacturing the laminates, molten silicon is infiltrated into the pre-impregnated lamina tapes to form a SiC and silicon mixed matrix.

The loading/unloading tensile experiments at room temperature were conducted on an MTS servo hydraulic load-frame (MTS System Crop., Minneapolis, MN, USA) equipped with edge-loaded grips, operated under displacement control with the loading rate of 0.1–0.5 mm/min. The gage-section strains were measured using a 25.4 mm clip-on MTS extensometer with a maximum displacement of 2% strain. The direct observations of matrix cracking were made using Mitutoyo binocular optical microscope (Tokyo, Japan). The matrix crack density was determined by counting the number of the cracks in a length of 5–10 mm.

## 3. Hysteresis Loops Models Considering Multiple Matrix Cracking Modes

**Figure 1:**
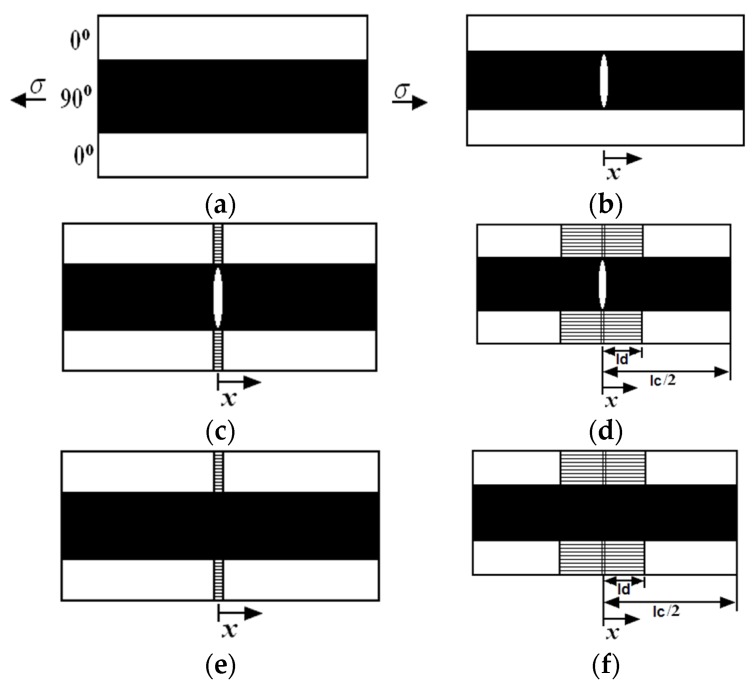
The undamaged state and five damaged modes of cross-ply ceramic composites: (**a**) undamaged composite; (**b**) mode 1: transverse crack; (**c**) mode 2: transverse crack and matrix crack with perfect fiber/matrix bonding; (**d**) mode 3: transverse crack and matrix crack with fiber/matrix interface debonding; (**e**) mode 4: matrix crack with perfect fiber/matrix bonding; and (**f**) mode 5: matrix cracking with fiber/matrix debonding.

**Figure 2:**
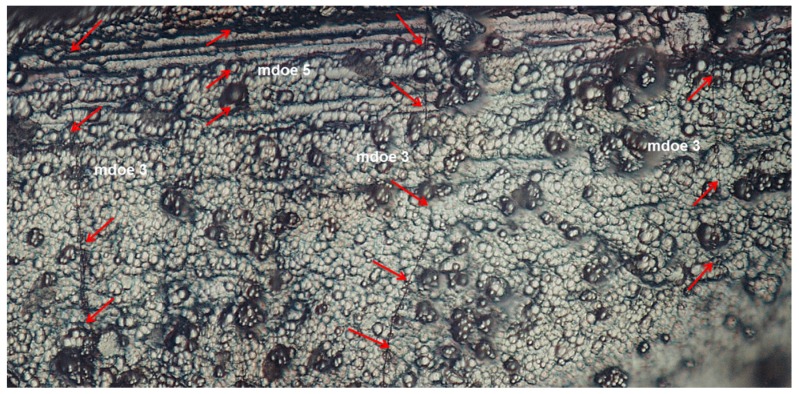
The matrix cracking mode 3 and mode 5 of cross-ply C/SiC composite under cyclic loading/unloading tensile.

Under cyclic loading, the matrix cracking modes in cross-ply CMCs can be divided into five different modes, *i.e.*, mode 1: transverse cracking in the 90° plies; mode 2: transverse cracking and matrix cracking occurred in the 90° and 0° plies, respectively, with perfect fiber/matrix interface bonding in the 0° plies; mode 3: transverse cracking and matrix cracking occurred in the 90° and 0° plies, respectively, with fiber/matrix interface debonding in the 0° plies; mode 4: matrix cracking in the 0° plies with fiber/matrix interface bonding; and mode 5: matrix cracking in the 0° plies with fiber/matrix interface debonding, as shown in Figure 1. Upon unloading and subsequent tensile reloading, matrix cracking mode 3 and mode 5 both exist within cross-ply C/SiC composite, as shown in Figure 2.

Upon unloading and reloading, the frictional slip occurred between fibers and the matrix in the 0° plies is the major reason for the hysteresis loops of cross-ply CMCs [5]. In cross-ply laminates, besides the fiber debonding and relative fiber/matrix sliding, other events, *i.e.*, delamination, relative ply sliding, near-tip matrix micro-cracking, and crack surface bridging followed by frictional fiber pull-out may also contribute to the hysteresis behavior. However, in the present analysis, the hysteresis loops models consider only the major factor of interface frictional slip in the matrix cracking mode 3 and mode 5. For matrix cracking mode 3, the hysteresis loops can be divided into four different cases, *i.e.*, case 1: interface partially debonds and fiber slips completely relative to matrix; case 2: interface partially debonds and fiber slips partially relative to matrix; case 3: interface completely debonds and fiber slips partially relative to matrix; and case 4: interface completely debonds and fiber slips completely relative to matrix. The unloading and reloading strains when interface partially debonds are [21]:

(1) $$\varepsilon_{\text{cu}}=\frac{\sigma }{V_{\text{f\_axial}}E_{f}}+4\frac{\tau_{i}}{E_{f}}\frac{y^{2}}{r_{f}l_{c}}-2\frac{\tau_{i}}{E_{f}}\frac{\left(2y-l_{d}\right)\left(2y-l_{c}+l_{d}\right)}{r_{f}l_{c}}-\left(\alpha_{c}-\alpha_{f}\right)\Delta \text{T}$$

(2) $$\begin{array}{l} \varepsilon_{\text{cr}}=\frac{\sigma }{V_{\text{f\_axial}}E_{f}}-4\frac{\tau_{i}}{E_{f}}\frac{z^{2}}{r_{f}l_{c}}+\frac{4\tau_{i}}{E_{f}}\frac{{\left(y-2z\right)}^{2}}{r_{f}l_{c}}\\ +2\frac{\tau_{i}}{E_{f}}\frac{\left(l_{d}-2y+2z\right)\left(l_{d}+2y-2z-l_{c}\right)}{r_{f}l_{c}}-\left(\alpha_{c}-\alpha_{f}\right)\Delta \text{T} \end{array}$$

in which *V*_f_axial_ denotes the fiber volume content in the 0° plies; *E*_f_ denotes the fiber elastic modulus; *r*_f_ denotes the fiber radius; *τ*_i_ denotes the fiber/matrix interface shear stress in the 0° plies; *l*_c_ denotes the matrix crack spacing; *l*_d_ denotes the interface debonded length; *α*_f_ and *α*_c_ denote the fiber and composite thermal expansion coefficient, respectively; ΔT denotes the temperature difference between fabricated temperature T_0_ and room temperature T_1_ (ΔT = T_1_ − T_0_); and *y* and *z* denote the interface counter-slip length and interface new-slip length, respectively.

When interface completely debonds, the unloading and reloading strains are [21]:

(3) $$\varepsilon_{\text{cu}}=\frac{\sigma }{V_{\text{f\_axial}}E_{f}}+4\frac{\tau_{i}}{E_{f}}\frac{y^{2}}{r_{f}l_{c}}-2\frac{\tau_{i}}{E_{f}}\frac{{\left(2y-l_{c}/2\right)}^{2}}{r_{f}l_{c}}-\left(\alpha_{c}-\alpha_{f}\right)\Delta \text{T}$$

(4) $$\varepsilon_{\text{cr}}=\frac{\sigma }{V_{\text{f\_axial}}E_{f}}-4\frac{\tau_{i}}{E_{f}}\frac{z^{2}}{r_{f}l_{c}}+4\frac{\tau_{i}}{E_{f}}\frac{{\left(y-2z\right)}^{2}}{r_{f}l_{c}}-2\frac{\tau_{i}}{E_{f}}\frac{{\left(l_{c}/2-2y+2z\right)}^{2}}{r_{f}l_{c}}-\left(\alpha_{c}-\alpha_{f}\right)\Delta \text{T}$$

For matrix cracking mode 5, the hysteresis loops can also be divided into four different cases. The unloading and reloading strains when interface partially debonds are [21]:

(5) $$\varepsilon_{\text{cu}}=\frac{1}{V_{\text{f\_axial}}E_{f}}\left(\sigma -k\sigma_{\text{to}}\right)+4\frac{\tau_{i}}{E_{f}}\frac{y^{2}}{r_{f}l_{c}}-2\frac{\tau_{i}}{E_{f}}\frac{\left(2y-l_{d}\right)\left(2y+l_{d}-l_{c}\right)}{r_{f}l_{c}}-\left(\alpha_{c}-\alpha_{f}\right)\Delta \text{T}$$

(6) $$\begin{array}{l} \varepsilon_{\text{cr}}=\frac{1}{V_{\text{f\_axial}}E_{f}}\left(\sigma -k\sigma_{\text{to}}\right)-4\frac{\tau_{i}}{E_{f}}\frac{z^{2}}{r_{f}l_{c}}+\frac{4\tau_{i}}{E_{f}}\frac{{\left(y-2z\right)}^{2}}{r_{f}l_{c}}\\ +2\frac{\tau_{i}}{E_{f}}\frac{\left(l_{d}-2y+2z\right)\left(l_{d}+2y-2z-l_{c}\right)}{r_{f}l_{c}}-\left(\alpha_{c}-\alpha_{f}\right)\Delta \text{T} \end{array}$$

in which *k* denotes the proportion of transverse plies in the entire composite.

When interface completely debonds, the unloading and reloading strains are [21]:

(7) $$\varepsilon_{\text{cu}}=\frac{1}{V_{\text{f\_axial}}E_{f}}\left(\sigma -k\sigma_{\text{to}}\right)+4\frac{\tau_{i}}{E_{f}}\frac{y^{2}}{r_{f}l_{c}}-2\frac{\tau_{i}}{E_{f}}\frac{{\left(2y-l_{c}/2\right)}^{2}}{r_{f}l_{c}}-\left(\alpha_{c}-\alpha_{f}\right)\Delta \text{T}$$

(8) $$\begin{array}{l} \varepsilon_{\text{cu}}=\frac{1}{V_{\text{f\_axial}}E_{f}}\left(\sigma -k\sigma_{\text{to}}\right)-4\frac{\tau_{i}}{E_{f}}\frac{z^{2}}{r_{f}l_{c}}+4\frac{\tau_{i}}{E_{f}}\frac{{\left(y-2z\right)}^{2}}{r_{f}l_{c}}\\ -2\frac{\tau_{i}}{E_{f}}\frac{{\left(l_{c}/2-2y+2z\right)}^{2}}{r_{f}l_{c}}-\left(\alpha_{c}-\alpha_{f}\right)\Delta \text{T} \end{array}$$

Considering the effect of multiple matrix cracking modes on hysteresis loops of cross-ply CMCs, the unloading and reloading strains of the composite are [21]:

(9) $${\left(\varepsilon_{u}\right)}_{c}=\eta {\left(\varepsilon_{\text{cu}}\right)}_{3}+\left(1-\eta \right){\left(\varepsilon_{\text{cu}}\right)}_{5}$$

(10) $${\left(\varepsilon_{r}\right)}_{c}=\eta {\left(\varepsilon_{\text{cr}}\right)}_{3}+\left(1-\eta \right){\left(\varepsilon_{\text{cr}}\right)}_{5}$$

in which (*ε*_u_)_c_ and (*ε*_r_)_c_ denote the unloading and reloading strain of the composite, respectively; (*ε*_cu_)_3_ and (*ε*_cr_)_3_ denote the unloading and reloading strain of the matrix cracking mode 3, respectively; (*ε*_cu_)_5_ and (*ε*_cr_)_5_ denote the unloading and reloading strain of the matrix cracking mode 5, respectively; *η* is the damage parameter determined by the composite’s damage condition, *i.e.*, the proportion of matrix cracking mode 3 in the entire of matrix cracking modes of the composite, *η* ∊ [0,1].

## 4. Experimental Comparisons

### 4.1. Cross-Ply C/SiC Composite

The cyclic loading/unloading tensile behavior of cross-ply C/SiC composite at room temperature has been investigated. The specimen was unloading and subsequent reloading at the peak stress of 20, 40, 60, 80, 100, and 120 MPa, respectively. The peak stress represents the macroscopic stress, *i.e.*, applied loading divided by the specimen cross section. The basic material properties of cross-ply C/SiC composite are given by: *V*_f_ = 40%, *E*_f_ = 230 GPa, *E*_m_ = 350 GPa, *r*_f_ = 3.5 μm, *τ*_i_ = 6 MPa, *ζ*_d_ = 0.1 J/m^2^, *α*_f_ = −0.38 × 10^−6^/°C, *α*_m_ = 2.8 × 10^−6^/°C, ΔT = −1000 °C.

**Figure 3:**
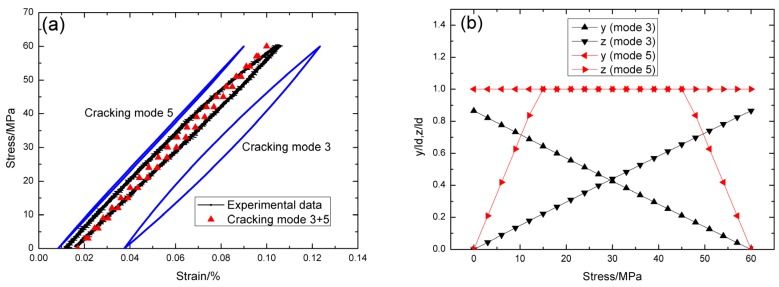
(**a**) The theoretical and experimental hysteresis loops; and (**b**) the interface slip lengths, *i.e.*, *y*/*l*_d_ and *z*/*l*_d_, of matrix cracking mode 3 and mode 5 of cross-ply C/SiC composite when *σ*_max_ = 60 MPa.

For *σ*_max_ = 60 MPa, the experimental and theoretical hysteresis loops are shown in Figure 3a, in which the proportion of matrix cracking mode 3 is *η* = 0.3. For matrix cracking mode 3, the hysteresis loops correspond to interface slip case 2, as shown in Figure 3b. Upon completely unloading, the interface counter-slip length approaches to 83.8% of interface debonded length, *i.e.*, *y*(*σ*_min_)/*l*_d_ = 86.5%, as shown in Figure 3b; upon reloading to peak stress, the interface new-slip length approaches to 86.5% of interface debonded length, *i.e.*, *z*(*σ*_max_)/*l*_d_ = 86.5%, as shown in Figure 3b. For matrix cracking mode 5, the hysteresis loops correspond to interface slip case 1, as shown in Figure 3b. Upon unloading, the interface counter-slip length approaches to interface debonded length at *σ*_tr_pu_ = 45 MPa, *i.e.*, *y*(*σ*_tr_pu_)/*l*_d_ = 1, as shown in Figure 3b; upon reloading to *σ*_tr_pr_ = 15 MPa, the interface new-slip length approaches to interface debonded length, *i.e.*, *z*(*σ*_tr_pr_)/*l*_d_ = 1, as shown in Figure 3b.

**Figure 4:**
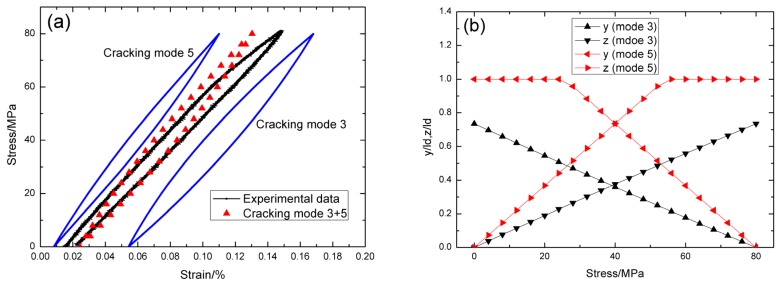
(**a**) The theoretical and experimental hysteresis loops; and (**b**) the interface slip lengths, *i.e.*, *y*/*l*_d_ and *z*/*l*_d_, of matrix cracking mode 3 and mode 5 of cross-ply C/SiC composite when *σ*_max_ = 80 MPa.

For *σ*_max_ = 80 MPa, the experimental and theoretical hysteresis loops are shown in Figure 4a, in which the proportion of matrix cracking mode 3 is *η* = 0.35. For matrix cracking mode 3, the hysteresis loops correspond to interface slip case 2, as shown in Figure 4b. Upon completely unloading, the interface counter-slip length approaches to 73.5% of interface debonded length, *i.e.*, *y*(*σ*_min_)/*l*_d_ = 73.5%, as shown in Figure 4b; upon reloading to peak stress, the interface new-slip length approaches to 73.5% of interface debonded length, *i.e.*, *z*(*σ*_max_)/*l*_d_ = 73.5%, as shown in Figure 4b. For matrix cracking mode 5, the hysteresis loops correspond to interface slip case 1, as shown in Figure 4b. Upon unloading, the interface counter-slip length approaches to interface debonded length at *σ*_tr_pu_ = 24 MPa, *i.e.*, *y*(*σ*_tr_pu_)/*l*_d_ = 1, as shown in Figure 4b; upon reloading to *σ*_tr_pr_ = 56 MPa, the interface new-slip length approaches to interface debonded length, *i.e.*, *z*(*σ*_tr_pr_)/*l*_d_ = 1, as shown in Figure 4b.

**Figure 5:**
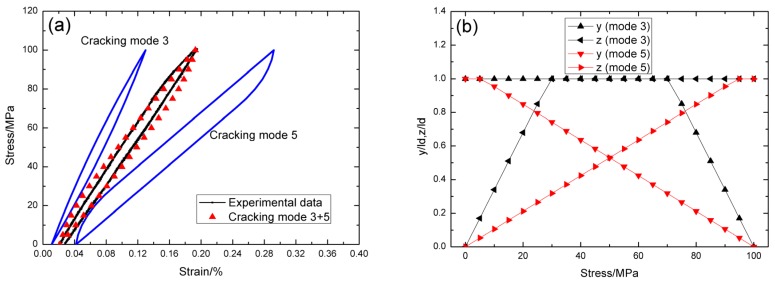
(**a**) The theoretical and experimental hysteresis loops; and (**b**) the interface slip lengths, *i.e.*, *y*/*l*_d_ and *z*/*l*_d_, of matrix cracking mode 3 and mode 5 of cross-ply C/SiC composite when *σ*_max_ = 100 MPa.

For *σ*_max_ = 100 MPa, the experimental and theoretical hysteresis loops are shown in Figure 5a, in which the proportion of matrix cracking mode 3 is *η* = 0.4. For matrix cracking mode 3, the hysteresis loops correspond to interface slip case 4, as shown in Figure 5b. Upon completely unloading, the interface counter-slip length approaches to matrix crack spacing at *σ*_tr_fu_ = 70 MPa, *i.e.*, 2*y*(*σ*_tr_fu_)/*l*_c_ = 1, as shown in Figure 5b; upon reloading to *σ*_tr_fr_ = 30 MPa, the interface new-slip length approaches to matrix crack spacing, *i.e.*, 2*z*(*σ*_tr_fr_)/*l*_c_ = 1, as shown in Figure 5b. For matrix cracking mode 5, the hysteresis loops correspond to interface slip case 1, as shown in Figure 5b. Upon unloading, the interface counter-slip length approaches to interface debonded length at *σ*_tr_pu_ = 95 MPa, *i.e.*, *y*(*σ*_tr_pu_)/*l*_d_ = 1, as shown in Figure 5b; upon reloading to *σ*_tr_pr_ = 5 MPa, the interface new-slip length approaches to interface debonded length, *i.e.*, *z*(*σ*_tr_pr_)/*l*_d_ = 1, as shown in Figure 5b.

**Figure 6:**
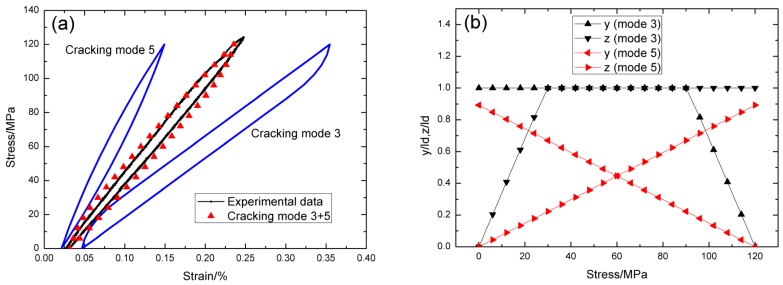
(**a**) The theoretical and experimental hysteresis loops; and (**b**) the interface slip lengths, *i.e.*, *y*/*l*_d_ and *z*/*l*_d_, of matrix cracking mode 3 and mode 5 of cross-ply C/SiC composite when *σ*_max_ = 120 MPa.

For *σ*_max_ = 120 MPa, the experimental and theoretical hysteresis loops are shown in Figure 6a, in which the proportion of matrix cracking mode 3 is *η* = 0.42. For matrix cracking mode 3, the hysteresis loops correspond to interface slip case 4, as shown in Figure 6b. Upon unloading, the interface counter-slip length approaches to matrix crack spacing at *σ*_tr_fu_ = 90 MPa, *i.e.*, 2*y*(*σ*_tr_fu_)/*l*_c_ = 1, as shown in Figure 6b; upon reloading to *σ*_tr_fr_ = 30 MPa, the interface new-slip length approaches to matrix crack spacing, *i.e.*, 2*z*(*σ*_tr_fr_)/*l*_c_ = 1, as shown in Figure 6b. For matrix cracking mode 5, the hysteresis loops correspond to interface slip case 2, as shown in Figure 6b. Upon completely unloading, the interface counter-slip length approaches to 89.3% of interface debonded length, *i.e.*, *y*(*σ*_min_)/*l*_d_ = 89.3%, as shown in Figure 6b; upon reloading to *σ*_max_ = 120 MPa, the interface new-slip length approaches to 89.3% of interface debonded length, *i.e.*, *z*(*σ*_max_)/*l*_d_ = 89.3%, as shown in Figure 6b.

### 4.2. Cross-Ply SiC/SiC Composite

Gordon [20] investigated the cyclic loading/unloading hysteresis behavior of cross-ply SiC/SiC composite. The loading/unloading peak stresses are 190, 200 and 210 MPa, respectively. The basic material properties of cross-ply SiC/SiC composite are given by reference [20]: *V*_f_ = 30%, *E*_f_ = 420 GPa, *E*_m_ = 364 GPa, *r*_f_ = 7.5 μm, *τ*_i_ = 15 MPa, *ζ*_d_ = 1.5 J/m^2^, *α*_f_ = 4.6 × 10^−6^/°C, *α*_m_ = 4.38 × 10^−6^/°C, and ΔT = −1400 °C.

**Figure 7:**
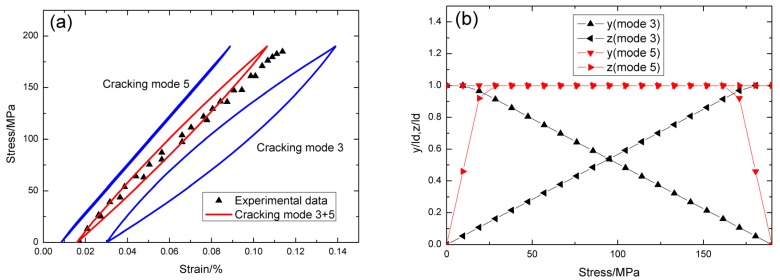
(**a**) The theoretical and experimental hysteresis loops; and (**b**) the interface slip lengths, *i.e.*, *y*/*l*_d_ and *z*/*l*_d_, of matrix cracking mode 3 and mode 5 of cross-ply SiC/SiC composite when *σ*_max_ = 190 MPa.

For *σ*_max_ = 190 MPa, the experimental and theoretical hysteresis loops are shown in Figure 7a, in which the proportion of matrix cracking mode 3 is *η* = 0.35. For matrix cracking mode 3, the hysteresis loops correspond to interface slip case 1, as shown in Figure 7b. Upon unloading, the interface counter-slip length approaches to interface debonded length at *σ*_tr_pu_ = 9.5 MPa, *i.e.*, *y*(*σ*_tr_pu_)/*l*_d_ = 1, as shown in Figure 7b; upon reloading to *σ*_tr_pr_ = 180.5 MPa, the interface new-slip length approaches to interface debonded length, *i.e.*, *z*(*σ*_tr_pr_)/*l*_d_ = 1, as shown in Figure 7b. For matrix cracking mode 5, the hysteresis loops correspond to interface slip case 1, as shown in Figure 7b. Upon unloading, the interface counter-slip length approaches to interface debonded length at *σ*_tr_pu_ = 161.5 MPa, *i.e.*, *y*(*σ*_tr_pu_)/*l*_d_ = 1, as shown in Figure 7b; upon reloading to *σ*_tr_pr_ = 28.5 MPa, the interface new-slip length approaches to interface debonded length, *i.e.*, *z*(*σ*_tr_pr_)/*l*_d_ = 1, as shown in Figure 7b.

**Figure 8:**
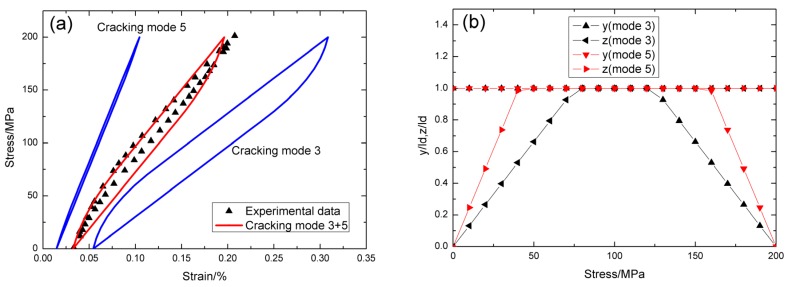
(**a**) The theoretical and experimental hysteresis loops; and (**b**) the interface slip lengths, *i.e.*, *y*/*l*_d_ and *z*/*l*_d_, of matrix cracking mode 3 and mode 5 of cross-ply C/SiC composite when *σ*_max_ = 200 MPa.

For *σ*_max_ = 200 MPa, the experimental and theoretical hysteresis loops are shown in Figure 8a, in which the proportion of matrix cracking mode 3 is *η* = 0.45. For matrix cracking mode 3, the hysteresis loops correspond to interface slip case 4, as shown in Figure 8b. Upon unloading, the interface counter-slip length approaches to matrix crack spacing at *σ*_tr_fu_ = 120 MPa, *i.e.*, 2*y*(*σ*_tr_fu_)/*l*_c_ = 1, as shown in Figure 8b; upon reloading to *σ*_tr_fr_ = 80 MPa, the interface new-slip length approaches to matrix crack spacing, *i.e.*, 2*z*(*σ*_tr_fr_)/*l*_c_ = 1, as shown in Figure 8b. For matrix cracking mode 5, the hysteresis loops correspond to interface slip case 1, as shown in Figure 8b. Upon unloading, the interface counter-slip length approaches to interface debonded length at *σ*_tr_pu_ = 150 MPa, *i.e.*, *y*(*σ*_tr_pu_)/*l*_d_ = 1, as shown in Figure 8b; upon reloading to *σ*_tr_pr_ = 50 MPa, the interface new-slip length approaches to interface debonded length, *i.e.*, *z*(*σ*_tr_pr_)/*l*_d_ = 1, as shown in Figure 8b.

**Figure 9:**
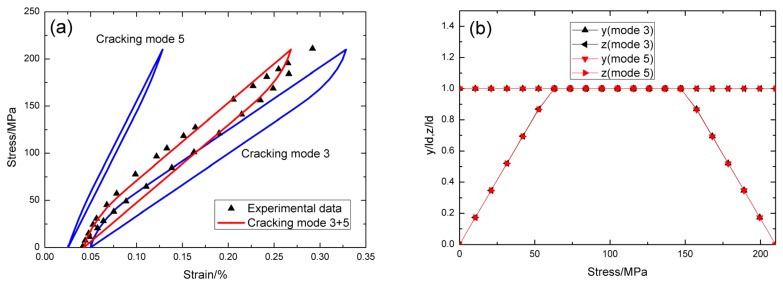
(**a**) The theoretical and experimental hysteresis loops; and (**b**) the interface slip lengths, *i.e.*, *y*/*l*_d_ and *z*/*l*_d_, of matrix cracking mode 3 and mode 5 of cross-ply SiC/SiC composite when *σ*_max_ = 210 MPa.

For *σ*_max_ = 210 MPa, the experimental and theoretical hysteresis loops are shown in Figure 9a, in which the proportion of matrix cracking mode 3 is *η* = 0.7. For matrix cracking mode 3, the hysteresis loops correspond to interface slip case 4, as shown in Figure 9b. Upon unloading, the interface counter-slip length approaches to matrix crack spacing at *σ*_tr_fu_ = 147 MPa, *i.e.*, 2*y*(*σ*_tr_fu_)/*l*_c_ = 1, as shown in Figure 9b; upon reloading to *σ*_tr_fr_ = 63 MPa, the interface new-slip length approaches to matrix crack spacing, *i.e.*, 2*z*(*σ*_tr_fr_)/*l*_c_ = 1, as shown in Figure 9b. For matrix cracking mode 5, the hysteresis loops correspond to interface slip case 1, as shown in Figure 9b. Upon unloading, the interface counter-slip length approaches to interface debonded length at *σ*_tr_pu_ = 147 MPa, *i.e.*, *y*(*σ*_tr_pu_)/*l*_d_ = 1, as shown in Figure 9b; upon reloading to *σ*_tr_pr_ = 63 MPa, the interface new-slip length approaches to interface debonded length, *i.e.*, *z*(*σ*_tr_pr_)/*l*_d_ = 1, as shown in Figure 9b.

## 5. Comparison between C/SiC and SiC/SiC Composite

**Figure 10:**
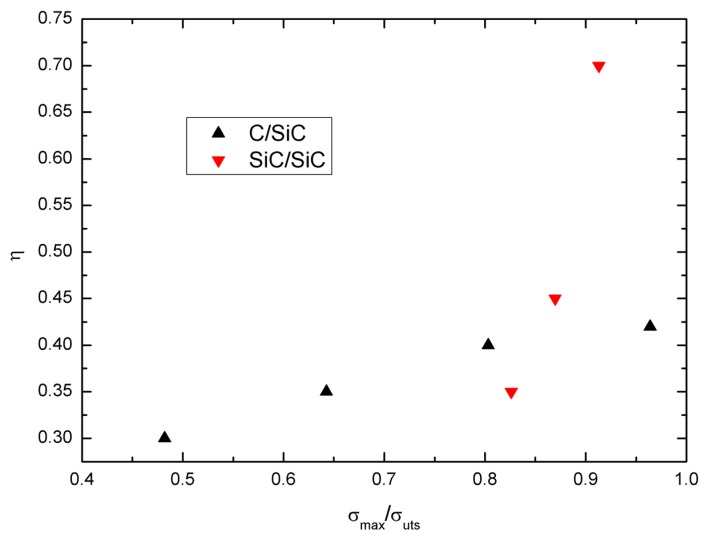
The damage parameter *η vs.* normalized stress *σ*_max_/*σ*_uts_ curves of cross-ply C/SiC and SiC/SiC composites.

**Figure 11:**
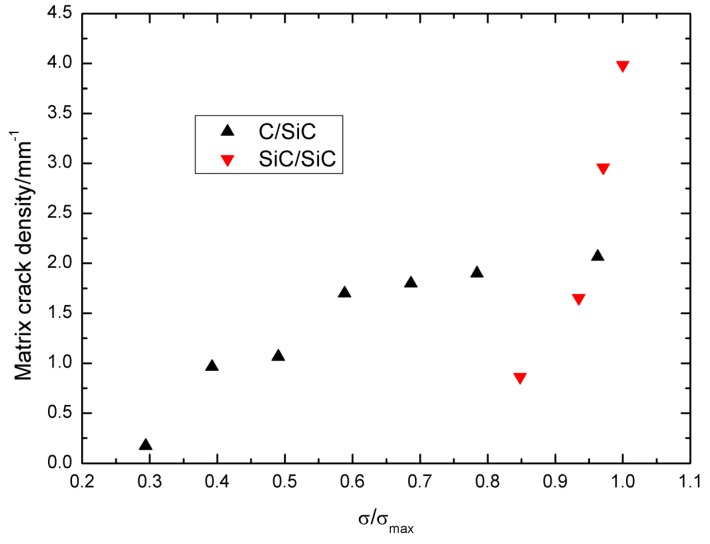
The matrix crack density of matrix cracking mode 3 in the 0° plies *vs.* normalized stress *σ*/*σ*_max_ of cross-ply C/SiC and SiC/SiC composites.

The damage parameter *η vs.* normalized stress *σ*_max_/*σ*_uts_ curves of cross-ply C/SiC and SiC/SiC composites are illustrated in Figure 10. With increasing peak stress, the damage parameter *η* increases. At low peak stress, the damage parameter *η* of cross-ply C/SiC is higher than that of cross-ply SiC/SiC composite, *i.e.*, *η* = 0.3 at *σ*_max_ = 60 MPa or 48.2% *σ*_uts_ of C/SiC composite, and *η* = 0.35 at *σ*_max_ = 190 MPa or 82.6% *σ*_uts_ of SiC/SiC composite. However, at high peak stress, the damage parameter *η* of cross-ply SiC/SiC is higher than that of cross-ply C/SiC composite, *i.e.*, *η* = 0.7 at *σ*_max_ = 210 MPa or 91.3% *σ*_uts_ of SiC/SiC composite, and *η* = 0.42 at *σ*_max_ = 120 MPa or 96.3% *σ*_uts_ of C/SiC composite. The matrix crack density of cracking mode 3 in the 0° plies *vs.* normalized stress *σ*/*σ*_max_ curves of cross-ply C/SiC and SiC/SiC composites can be used to show the damage extent inside of composites, as shown in Figure 11. It can be found that the matrix crack density of cracking mode 3 in the C/SiC composite is higher than that of SiC/SiC composite under lower peak stress, *i.e.*, *σ*/*σ*_max_ < 0.8, and lower for C/SiC composite than that of SiC/SiC composite under higher peak stress, *i.e.*, *σ*/*σ*_max_ > 0.8.

As the axial thermal residual tensile stress existed in SiC matrix due to the large mismatch of the axial thermal expansion coefficient between carbon fibers and silicon carbide matrix, *i.e.*, −0.38 × 10^−6^/°C *vs*. 2.8 × 10^−6^/°C, and the radial thermal residual tensile stress existed in the fiber/matrix interface due to the large mismatch of the radial thermal expansion coefficient between carbon fibers and silicon carbide matrix, *i.e.*, 7 × 10^−6^/°C *vs.* 2.8 × 10^−6^/°C, there are unavoidable microcracks existing within the SiC matrix in the 90° and 0° plies when the composite was cooled down from high fabricated temperature to ambient temperature. These processing-induced microcracks propagated and, in conjunction with new microcracks during the loading process, formed mode 5 matrix cracks in the 90° plies. With increasing applied stress, some matrix cracks in the 90° plies connected with matrix cracks in the 0° plies forming mode 3 matrix cracks, which propagate through the 90° and 0° plies. For cross-ply SiC/SiC composite, the axial thermal residual compressive stress existed in SiC matrix due to the large mismatch of the axial thermal expansion coefficient between silicon carbide fibers and silicon carbide matrix, *i.e.*, 5.1 × 10^−6^/°C *vs.* 3.5 × 10^−6^/°C, and the radial thermal residual compressive stress existed in the fiber/matrix interface due to the large mismatch of the radial thermal expansion coefficient between silicon carbide fibers and silicon carbide matrix, *i.e.*, 2.9 × 10^−6^/°C *vs.* 3.5 × 10^−6^/°C, which decreases matrix cracking evolution rate and also the damage parameter *η* at low peak stress. However, with increasing peak stress, the damage extent inside of cross-ply SiC/SiC composite, *i.e.*, the damage parameter *η*, is much higher than that of C/SiC composite as more transverse cracks and matrix cracks connecting together.

**Figure 12:**
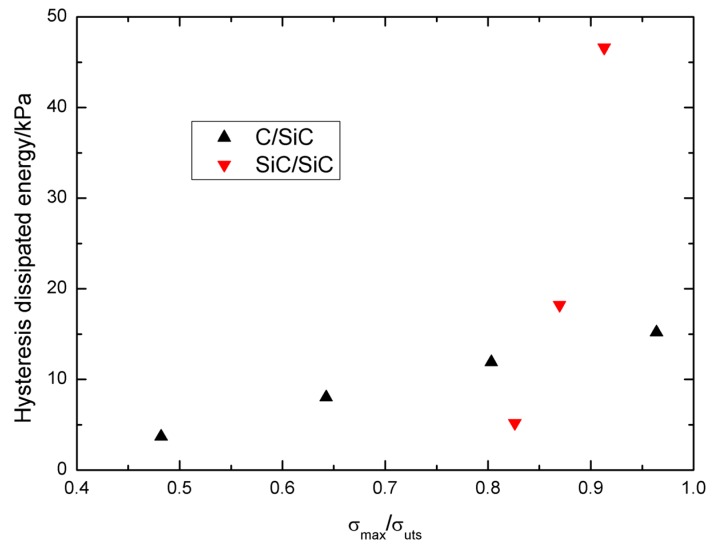
The hysteresis dissipated energy *vs.* normalized stress *σ*_max_/*σ*_uts_ curves of cross-ply C/SiC and SiC/SiC composites.

The hysteresis dissipated energy *vs.* normalized stress *σ*_max_/*σ*_uts_ curves of cross-ply C/SiC and SiC/SiC composites are illustrated in Figure 12. With increasing peak stress, the hysteresis dissipated energy of C/SiC and SiC/SiC composites increase, *i.e.*, from 3.7 kPa at *σ*_max_ = 60 MPa or 48.2% *σ*_uts_, to 15.2 kPa at *σ*_max_ = 120 MPa or 96.3% *σ*_uts_; and from 5.2 kPa at *σ*_max_ = 190 MPa or 82.6% *σ*_uts_, to 46.6 kPa at *σ*_max_ = 210 MPa or 91.3% *σ*_uts_. The hysteresis dissipated energy of C/SiC composite under low peak stress is higher than that of SiC/SiC composite due to a higher damage parameter *η* at low peak stress of C/SiC composite compared with that of SiC/SiC composite. However, at high peak stress, the damage parameter *η* of SiC/SiC composite is higher than that of C/SiC composite, leading to higher hysteresis dissipated energy compared with that of C/SiC composite.

For C/SiC composite, the hysteresis loops of matrix cracking mode 3 and mode 5 correspond to interface slip case 2 and case 1, respectively, under low peak stresses of 60 and 80 MPa; when the peak stress is 100 MPa, the hysteresis loops of matrix cracking mode 3 transfers from case 2 to case 4; and when the peak stresses are 120 MPa, the hysteresis loops of matrix cracking mode 5 transfers from case 1 to case 2, as shown in Table 1. For SiC/SiC composite, the hysteresis loops of matrix cracking mode 3 and mode 5 both correspond to interface slip case 1 under low peak stresses of 190 MPa; and when the peak stress increases to 200 MPa, the hysteresis loops of matrix cracking mode 3 transfers from case 1 to case 4, and the hysteresis loops of matrix cracking mode 5 remains to be interface slip case 1, as shown in Table 2.

**Table 1:** The interface slip type of matrix cracking mode 3 and mode 5 corresponding to different peak stresses of cross-ply C/SiC composite.

Cracking Modes	60 MPa	80 MPa	100 MPa	120 MPa
Matrix cracking mode 3	case 2	case 2	case 4	case 4
Matrix cracking mode 5	case 1	case 1	case 1	case 2

**Table 2:** The interface slip type of matrix cracking mode 3 and mode 5 corresponding to different peak stresses of cross-ply SiC/SiC composite.

Cracking Modes	190 MPa	200 MPa	210 MPa
Matrix cracking mode 3	case 1	case 4	case 4
Matrix cracking mode 5	case 1	case 1	case 1

## 6. Conclusions

The comparison of cyclic hysteresis behavior between cross-ply C/SiC and SiC/SiC CMCs has been investigated. The interface slip between fibers and the matrix existed in matrix cracking mode 3 and mode 5 are considered as the major reason for hysteresis loops of cross-ply CMCs. The hysteresis loops of cross-ply C/SiC and SiC/SiC composites corresponding to different peak stresses have been predicted using present analysis. The differences between C/SiC and SiC/SiC composite on damage parameters and hysteresis dissipated energy have been investigated.

(1) The damage parameter, *i.e.*, the proportion of matrix cracking mode 3 in the entire matrix cracking modes of the composite, and the hysteresis dissipated energy both increase with increasing peak stress;
(2) The damage parameter and hysteresis dissipated energy of C/SiC composite under low peak stress are higher than those of SiC/SiC composite; However, with increasing peak stress, the damage extent inside of cross-ply SiC/SiC composite, *i.e.*, the damage paramter *η* and hysteresis dissipated energy, is much higher than that of C/SiC composite as more transverse cracks and matrix cracks connecting together.

## References

[1] Naslain R. (2004). Design, preparation and properties of non-oxide CMCs for application in engines and nuclear reactors: An overview. Compos. Sci. Technol..

[2] Bednarcyk B.A., Mital S.K., Pineda E.J., Arnold S.M. Multiscale modeling of ceramic matrix composites. Proceedings of the 56th AIAA/ASCE/AHS/ASC Structures Dynamics Materials Conference.

[3] Gowayed Y., Ojard G., Santhosh U., Jefferso G. (2015). Modeling of crack density in ceramic matrix composites. J. Compos. Mater..

[4] Reynaud P. (1996). Cyclic fatigue of ceramic-matrix composites at ambient and elevated temperatures. Compos. Sci. Technol..

[5] Fantozzi G., Reynaud P. (2009). Mechanical hysteresis in ceramic matrix composites. Mater. Sci. Eng. Part A Struct..

[6] Kotil T., Holmes J.W., Comninou M. (1990). Origin of hysteresis observed during fatigue of ceramic matrix composites. J. Am. Ceram. Soc..

[7] Pryce A.W., Smith P.A. (1993). Matrix cracking in unidirectional ceramic matrix composites under quasi-static and cyclic loading. Acta Metall. Mater..

[8] Ahn B.K., Curtin W.A. (1997). Strain and hysteresis by stochastic matrix cracking in ceramic matrix composites. J. Mech. Phys. Solids.

[9] Solti J.P., Mall S., Robertson D.D. (1995). Modeling damage in unidirectional ceramic-matrix composites. Compos. Sci. Technol..

[10] Vagaggini E., Domergue J.M., Evans A.G. (1995). Relationships between hysteresis measurements and the constituent properties of ceramic matrix composites: I, theory. J. Am. Ceram. Soc..

[11] Hutchison J.W., Jensen H.M. (1990). Models of fiber debonding and pullout in brittle composites with friction. Mech. Mater..

[12] Cho C.D., Holmes J.W., Barber J.R. (1991). Estimation of interfacial shear in ceramic composites from frictional heating measurements. J. Am. Ceram. Soc..

[13] Li L.B., Song Y.D., Sun Z.G. (2009). Influence of interface de-bonding on the fatigue hysteresis loops of ceramic matrix composites. Chin. J. Solid. Mech..

[14] Li L.B., Song Y.D., Sun Z.G. (2009). Effect of fiber Poisson contraction on fatigue hysteresis loops of ceramic matrix composites. J. Nanjing Univ. Aero. Astron..

[15] Li L.B., Song Y.D. (2011). Influnece of fiber failure on fatigue hysteresis loops of ceramic matrix composites. J. Reinf. Plast. Compos..

[16] Li L.B. (2015). Modeling the effect of interface wear on fatigue hysteresis behavior of carbon fiber-reinforced ceramic-matrix composites. Appl. Compos. Mater..

[17] Li L.B., Song Y.D., Sun Y.C. (2013). Estimate interface shear stress of unidirectional C/SiC ceramic matrix composites from hysteresis loops. Appl. Compos. Mater..

[18] Kuo W.S., Chou T.W. (1995). Multiple cracking of unidirectional and cross-ply ceramic matrix composites. J. Am. Ceram. Soc..

[19] (2015). Standard Practice for Constant-Amplitude, Axial, Tension-Tension Cyclic Fatigue of Continuous Fiber-Reinforced Advanced Ceramics at Ambient Temperatures.

[20] Gordon N. (2014). Material Health Monitoring of SiC/SiC Laminated Ceramic Matrix Composites With Acoustic Emission And Electrical Resistance. Master Thesis.

[21] Li L.B., Song Y.D., Sun Y.C. (2014). Effect of matrix cracking on hysteresis behavior of cross-ply ceramic matrix composites. J. Compos. Mater..

